# Evaluating the Correlation between Eyeglass-Type Wearable Device Measurements and Subjective Physical Activity Assessments

**DOI:** 10.7759/cureus.67853

**Published:** 2024-08-26

**Authors:** Masahiro Inoue, Shiro Sugiura, Taiki Takeda, Takato Hoshino, Keisuke Shimizu, Kazuhide Inage, Yasuhiro Shiga, Kohei Okuyama, Seiji Ohtori, Sumihisa Orita

**Affiliations:** 1 Department of Orthopaedic Surgery, Graduate School of Medicine, Chiba University, Chiba, JPN; 2 Rehabilitation, Nishikawa Orthopaedic Clinic, Sakura, JPN; 3 Center for Frontier Medical Engineering, Chiba University, Chiba, JPN; 4 Future Medicine Education and Research Organization, Chiba University, Chiba, JPN; 5 Orthopaedics, Chiba University Hospital, Chiba, JPN

**Keywords:** walking speed, metabolic equivalents, physical activity, international physical activity questionnaire, eyeglass-type wearable device

## Abstract

Introduction Wearable trackers are instrumental in monitoring various health indicators, notably daily physical activity, which is crucial for managing chronic diseases and improving overall health. This study examined the relationship between physical activity levels measured using JINS MEME, an eyeglass-type wearable device equipped with motion sensors, and subjective activity assessments reported through the International Physical Activity Questionnaire (IPAQ).

Methods Healthy volunteers aged 20-60 were recruited for an observational study. Participants wore the JINS MEME throughout the day for one week, and data on walking activity were collected and analyzed alongside IPAQ responses to evaluate subjective physical activity levels. The correlation between the two sets of data was evaluated using the nonparametric Spearman’s rho (ρ) correlation coefficient for both the assessed metabolic equivalents (METs) score of the JINS MEME and the IPAQ. Similarly, the relationship between the IPAQ questionnaire items and the measurements from the JINS MEME.

Results The study included 42 participants and revealed a strong correlation (R=0.719, P<0.01) between the metabolic equivalents (METs) calculated from the JINS MEME and IPAQ scores, especially for walking activities. Similarly, a significant association was found between the IPAQ data and walking speed (R=0.129, P=0.02). METs showed significant relationships with all physical activities, except sitting or reclining time.

Conclusion This study validated the use of eyeglass-type wearable devices, such as the JINS MEME, to accurately assess physical activity levels, demonstrating a strong correlation with subjective assessments using the IPAQ. This highlights the potential of wearable devices in comprehensive health monitoring and management strategies.

## Introduction

Recent advancements in wearable technology have significantly transformed the methods of collecting and analyzing health-related data in everyday life [1]. Notably, the smartwatch has been reported to detect atrial fibrillation during regular use, highlighting the potential of these devices for health monitoring [2]. Wearable devices play a crucial role in tracking a wide range of health indicators, including daily physical activity levels, which are essential for managing chronic diseases and enhancing overall health [3].

Our research group previously identified a correlation between the intensity of low back pain and physical activity levels as measured using a wristwatch-based device [4,5]. Furthermore, existing literature has shown a moderate correlation between the data obtained from wristwatch-type devices and self-reported activity levels, according to the International Physical Activity Questionnaire (IPAQ) [6]. The IPAQ is a validated self-reported physical activity measure used internationally for adults aged 15-69 years [7]. This suggests that wearable technology is a reliable tool for quantifying physical activity.

However, there is a noticeable gap in the research regarding the tracking of head movements, with most studies focusing on the movements of the arms, legs, and torso [8,9]. The evaluations of physical activity recognition and energy expenditure are most commonly conducted at the hip and wrist locations [10]. While some research has been conducted on monitoring physical activity using wearable devices such as eSense and sensors embedded in headbands, there is little research on activity levels using eyeglass-type wearable devices [11,12].

Glasses are widely used across various age groups, and the need for continuous wear facilitates consistent data collection. Given the widespread use of eyeglass-type devices, their potential for further development in health monitoring is significant. The current study sought to explore the relationship between physical activity levels measured using eye-wear-based wearable devices and subjective activity assessments reported through the IPAQ.

## Materials and methods

Participants and ethical considerations

This was an observational study. The subjects were healthy volunteers aged 20-60 years. The number of subjects was approximately 40, based on past literature and previous validation studies we conducted using wearable devices [8,13,14]. To analyze their movements in daily life and conduct a questionnaire survey, participants with any difficulty in walking, mental illness, Obesity with a BMI greater than 30, or cognitive impairment were excluded. In this study, non-prescription JINS MEME were used due to their universal applicability. Consequently, participants who normally require prescription eyeglasses were either instructed to use their personal contact lenses during the periods when JINS MEME were worn or were excluded from the study. This study was approved by the Ethics Committee of our institution. All participants were informed of the purpose of the study, received information, and provided written consent.

Analysis of physical activity using the JINS MEME

**Figure 1:**
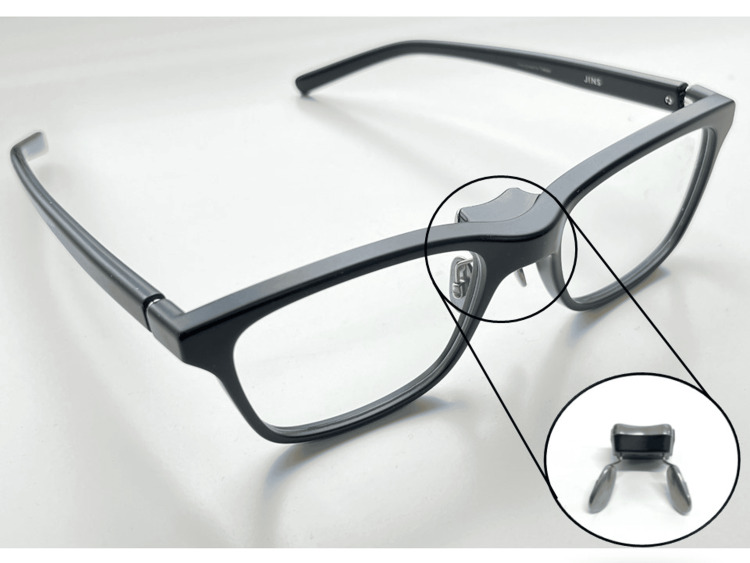
The JINS MEME with a built-in gyroscope and acceleration sensor electrodes This figure is an original image. Exterior view of the JINS MEME. The JINS MEME has a shape similar to that of glasses used in daily life. The circle on the nosepiece of the glasses is the core. The core contains Bluetooth, a battery, a 6-axis motion sensor, and an ocular electrode sensor.

The JINS MEME (JINS Inc., Tokyo) are commercially available glasses with a built-in gyroscope and acceleration sensor electrodes (Figure 1). The 6-axis motion sensor (3-axis acceleration sensor and 3-axis gyro sensor) mounted on the device can detect body movements and collect real-time data [15-17]. In this study, participants wore the JINS MEME from the time they woke up to the time they went to bed, and their data were measured. The data collection period was one week.

The following data indices that could be measured with the JINS MEME were used: tl_xav = Mean tilt X (degree), tl_yav = Mean tilt Y (degree), tl_yav = Mean tilt Y (degree), tl_yav = Mean tilt Y (degree), and tl_yav = Mean tilt Y (degree). Mean tilt Y (degree), stp_s = Walking seconds(s), stp_fst, stp_mid, stp_slw, and stp_vsl are Step count (High 280-370 ms), Step count (Mid 380-440 ms), Step count (Low 450-590 ms), and Step count (Extra-low 600-1000 ms), respectively.

Data extraction was performed using these indices according to the following protocol.

tl_xav > -45, tl_xav < 45, tl_yav > -45, tl_yav < 90 (filtered for wearing glasses)

stp_s >= 50 (only data with at least 50 seconds of walking per minute are extracted)

Mean((stp_fst + stp_mid + stp_slw + stp_vsl) * 60/stp_s) (Number of steps walked in 60 seconds was calculated) This was defined as walking speed.

Based on previous studies, the relationship between walking speed and metabolic equivalents (METs) for 60 seconds was defined as follows: [18]

90 steps or less = 2 METs, 91-100 steps = 3 METs, 101-110 steps = 4 METs, 111-120 steps = 5 METs, 121 steps or more = 6 METs Total METs per week were calculated for comparison with IPAQ.

The physical activity evaluated using IPAQ

The Japanese version of IPAQ was used in this study. Also, the IPAQ is available in two versions: long and short. The short version of the questionnaire was used to evaluate the total amount of physical activity rather than the amount of physical activity for each task.

The following values were used for the analysis of IPAQ data: Walking = 3.3 METs, Moderate PA = 4.0 METs and Vigorous PA = 8.0 METs. [19] Total MET-minutes/week = Walk (METs*min*days) + Mod (METs*min*days) + Vig (METs*min*days)

To compare the data obtained by wearing the JINS MEME with the IPAQ scores, the participants were asked about their physical activity while wearing the JINS MEME at the end of data collection.

Statistical analysis

The correlation between the two sets of data was evaluated using the nonparametric Spearman’s rho (ρ) correlation coefficient for both the assessed METS score of the JINS MEME and the IPAQ. Similarly, the association between walking speed and METs in the IPAQ was evaluated. Furthermore, the relationship between the IPAQ questionnaire items (vigorous, moderate, walking, and sitting time) and the measurements from the JINS MEME was assessed using the nonparametric Spearman’s rho (ρ) correlation coefficient to determine which physical activities were more closely related to head movements. Statistical significance was set at P<0.05. All statistical procedures were performed using JMP® 15 software (SAS Institute Inc., Cary, NC, USA). All data are reported as means ± standard deviations unless otherwise indicated.

## Results

The current study included 42 participants (35 men, 7 women). Demographic data, including data measured using the JINS MEME and analyzed using the IPAQ, are presented in Table 1. Among the metrics measured using the JINS MEME, the average walking speed was 111.6±7.4 steps/min, and the average total METs was 925.4±655.1 METs/week. In the IPAQ data, the time spent in vigorous, moderate, and walking activities, as well as sitting and reclining time per week, were 78.7 ± 116.2, 62.0 ± 88.9, 251.6 ± 231.4, and 359.2±270.5 minutes, respectively, with the average total METs being 1766.5 ± 1498.2 METs/week.

**Table 1:** Demographic data

No, of participants	42
Age, mean (range), years	35.2 ± 8.6 (21-52)
Gender (Male/female)	35/7
Body Mass Index (kg/m^2^)	23.0 ± 3.1
JINS MEME data	
Walking speed (/min)	111.6 ± 7.4
METs (/week)	925.4 ± 655.1
IPAQ data	
Vigorous (min/week)	78.7 ± 116.2
Moderate (min/week)	62.0 ± 88.9
Walking (min/week)	251.6 ± 231.4
Sitting or reclining (min/week)	359.2 ± 270.5
METs (/week)	1766.5 ± 1498.2

**Figure 2:**
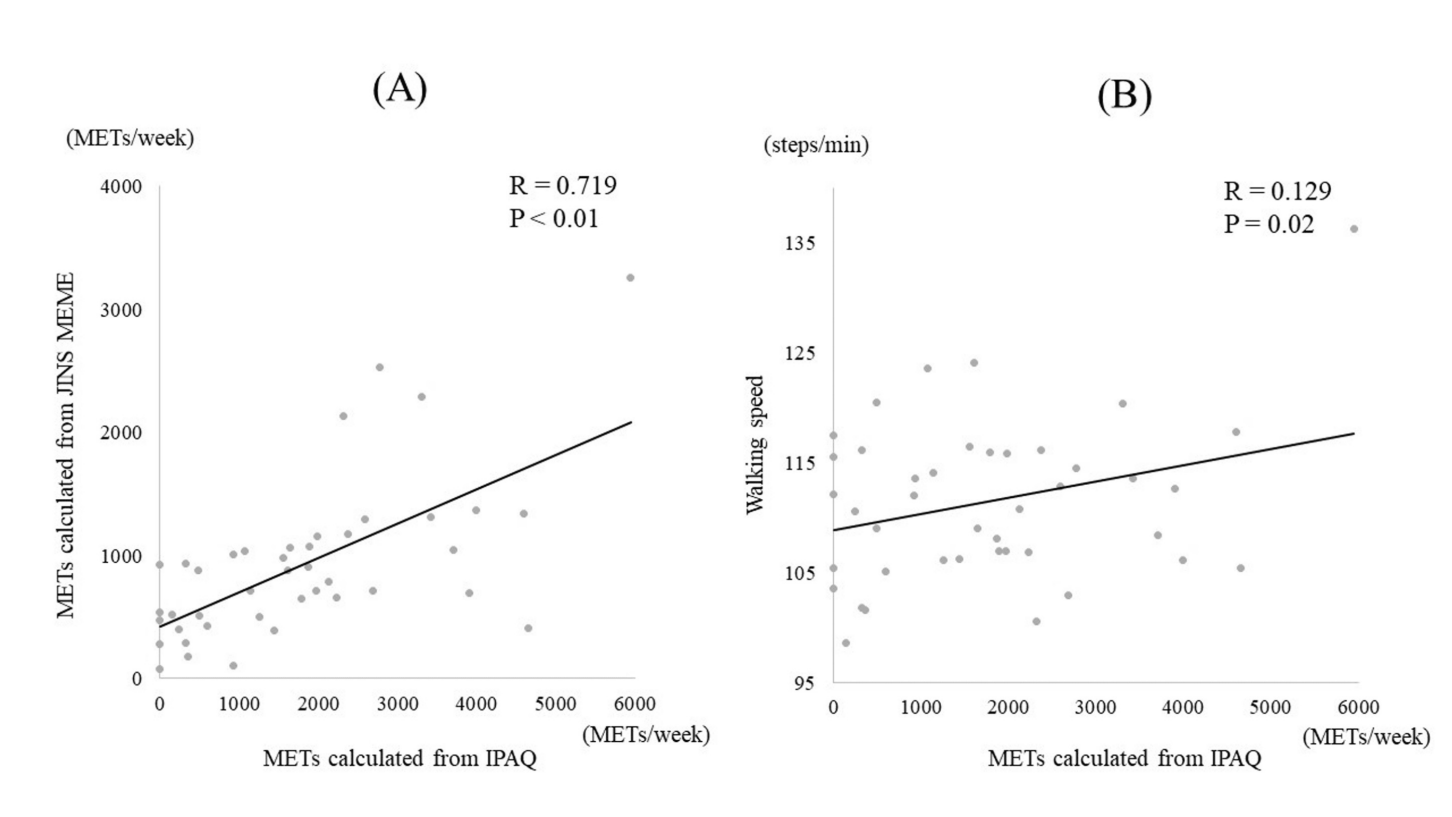
The correlation between the score of the JINS MEME and the IPAQ Graph (A) shows the correlation between METs calculated from JINS MEME and METs calculated from IPAQ; there was a strong correlation between METs calculated from JINS MEME and METs calculated from IPAQ. Graph (B) shows the correlation between walking speed measured by JINS MEME and METs calculated from IPAQ. A correlation was also found between walking speed and METs calculated from IPAQ. METs: metabolic equivalents; IPAQ: International Physical Activity Questionnaire

A strong correlation (R=0.719, P<0.01) was observed between the METs calculated from the JINS MEME measurements and IPAQ. Similarly, a significant association was found between the IPAQ data and walking speed (R=0.129, P=0.02) (Figure 2).

When examining the data from the JINS MEME in relation to the individual items of the IPAQ, no association was observed between walking speed and individual items. However, a significant relationship was found between METs and all items, except sitting or reclining time, with a particularly strong correlation noted for walking (Table 2).

**Table 2:** Relationship between JINS MEME data and IPAQ individual items IPAQ: International Physical Activity Questionnaire; METs: metabolic equivalents

		JINS MEME			
		Walking speed		METs	
		Correlation coefficient	p-value	Correlation coefficient	p-value
IPAQ	Vigorous	0.019	0.904	0.351	0.025
	Moderate	0.076	0.636	0.318	0.043
	Walking	0.262	0.098	0.613	< 0.01
	Sitting or reclining	0.075	0.642	0.222	0.164

## Discussion

In this study, we evaluated the correlation between physical activity measured using eyeglass-type wearable devices and patient-reported outcomes assessed using physical activity levels. Our findings revealed a strong correlation between the data measured using the JINS MEME and IPAQ scores. Notably, this correlation was particularly strong for the walking data, which was evident in both the measurements and questionnaire scores.

Several studies have highlighted the correlation between wearable device measurements and survey assessments. Jelsma et al. demonstrated the relationship between post-THA activity levels measured using wearable devices and physical activity assessed through questionnaires [20]. However, discrepancies have been reported regarding the correlation between wearable device-measured activity levels and questionnaire-assessed improvement after spinal surgery [21]. Our study supports the feasibility of using the JINS MEME to calculate physical activity levels, as indicated by its strong correlation with IPAQ scores.

The JINS MEME, a wearable accelerometer, showed a strong correlation with walking, suggesting its effectiveness in evaluating dynamic activities such as yoga and sports. Chen and Epps reported that head movement analysis using wearable sensors accurately recognizes four dimensions of task load, including cognitive, perceptual, communicative, and physical [22]. However, measuring activities that do not involve head movements, such as exercises performed in a stationary position or lifting weights, may be challenging. This limitation was evident in our study, where discrepancies were observed between IPAQ scores and JINS MEME measurements in participants who regularly engaged in upper limb and core strength training.

Several reports have addressed issues associated with wearable device placement. Davoudi et al. performed an experiment using six different wear positions (upper arm, wrist, hip, thigh, and ankle) and found that the hip position was the best for estimating both physical activity and energy expenditure [9]. On the other hand, Hildebrand et al. investigated the impact of device placement on the accelerometer output and found significant differences between wrist and hip placement [23]. There are also portability and reliability issues. Smartphones, which cannot always be carried, may lead to the underestimation of activity levels. [24] Continuity issues have also been reported among individuals with disabilities and the elderly [25].

However, eyeglass-type devices, which are wearable throughout most daily activities, have a high potential for constant use among diverse populations, including the elderly and disabled. Eyeglasses are a common accessory, with significant usage rates among adults both in Europe (60.3%) and Singapore (63.4%), primarily for vision correction [26,27]. In the United States, more than 93 million US adults (37.9%) were at high risk for vision loss; many adults used eye care in 2017 [28]. With the prevalence of myopia, especially in East Asia, and an estimated 5 billion people affected globally by 2050, the population using eyeglasses is expected to increase, highlighting the utility of eyeglasses as a consistently wearable device [29]. Certainly, compliance issues may arise for those who are not accustomed to wearing glasses when using the JINS MEME. Unfamiliarity with glasses could lead to discomfort and inconsistent use, which might affect the reliability and continuity of data collection. Therefore, improving the wearability and comfort of such wearable devices should be the focus of ongoing enhancements.

The current study had several limitations. First, the small sample size and gender imbalance necessitate further evaluations with larger datasets, including participants of various ages, health conditions, and lifestyles. Moreover, future studies should also explore conditions such as locomotive syndrome and sarcopenia, which are related to walking speed. Additionally, this study focused on the correlation with the IPAQ, a subjective assessment, and thus did not precisely evaluate individual METs. Because our activity measurements were based on walking speed and step count, non-ambulatory activities such as lifting weights may have been underestimated.

Although there are limitations to the measurement environment and exercise content, the JINS MEME, an eyeglass-type wearable device, has shown a strong correlation with the IPAQ in activity measurement and is useful in measuring daily activities.

Future scope of the study

Future research should explore methods that effectively measure both static exercises with minimal head movement and dynamic activities such as walking to accurately capture a broader range of activities. Additionally, future evaluations should consider utilizing devices like pedometers and accelerometers to determine which types and locations of measurement most accurately reflect physical activity levels. Furthermore, expanding the sample size and diversity of study participants is critical to generalizing these findings and increasing the applicability of wearable technology across different demographics and lifestyles. Future studies should also consider the application of this technology to chronic diseases and age-related conditions such as locomotive syndrome and sarcopenia.

## Conclusions

This study demonstrates that the JINS MEME, an eyeglass-type wearable device, is effective in measuring physical activity levels and strongly correlates with IPAQ scores, as reported by conventional questionnaires. The association between measurements from wearable device and self-reported physical activity questionnaires highlights the potential not only to increase the accuracy of health assessments but also to expand the scope of monitoring to include a wider range of activities that are often overlooked by traditional methods.

However, the study also highlighted challenges in tracking non-ambulatory activities that do not involve significant head movements, such as weight lifting and static exercise. This limitation suggests that while wearable devices like the JINS MEME are useful for capturing a wide range of physical activity, further improvements are needed to accurately measure all forms of physical exertion.

The integration of eyeglass-type devices into health monitoring has great potential due to their continuous portability and widespread use. As the global population of eyeglass users is expected to increase, particularly with the increasing prevalence of conditions such as myopia, the use of these devices for health monitoring is likely to be very useful not only for individuals but also for public health.
